# Blind Rush? Shale Gas Boom Proceeds Amid Human Health Questions

**DOI:** 10.1289/ehp.119-a348

**Published:** 2011-08-01

**Authors:** Charles W. Schmidt

**Affiliations:** Charles W. Schmidt, MS, an award-winning science writer from Portland, ME, has written for *Discover Magazine*, *Science*, and *Nature Medicine*.

In 2003 Range Resources, a natural gas company based in Fort Worth, Texas, was among the first of its competitors working on what appeared to be a promising deposit in Pennsylvania called the Marcellus Shale. Geologists had long believed the Marcellus was full of gas trapped in shale pores deep underground, like bubbles in fossilized soda. Range Resources was hoping to tap it with a method called hydraulic fracturing (or fracking for short) in which a high-volume mixture of water, sand, and chemicals is pumped into the shale under pressure. Several companies had used this approach to liberate natural gas from the Barnett Shale, a similar formation in Texas.

When Range Resources fracked the Marcellus Shale, the yields were at first modest. So the company shifted its approach; instead of drilling vertical wells straight into the shale, they drilled wells that could also turn sideways thousands of feet below the surface and then probe horizontally for miles in any direction. With horizontal drilling, the yields got steadily better, until Range Resources hit a jackpot in 2006: a gas-rich formation that might generate 50 years of profits for the company, according to spokesman Matt Pitzarella. That discovery helped confirm that the Marcellus—which cuts across portions of at least eight eastern states from New York to Tennessee—is one of the largest shale gas deposits in the world. A $400 million company in 2003, Range Resources is now valued at more than $8 billion, largely because of its Marcellus lease holdings.^1^

Meanwhile, the combination of fracking and horizontal drilling has sent potentially recoverable amounts of natural gas nationwide soaring. The Energy Information Administration estimates that technically recoverable shale gas reserves have the potential to satisfy domestic consumption in the United States (based on 2010 figures^2^) for more than 30 years.^3^

But for shale gas to meet its potential, millions of Americans will have to live with drill rigs in or near their own neighborhoods. And that opens the door to a range of potential environmental health problems: pipelines and wellheads can explode, the process produces toxic air emissions, and fracking generates liquid wastes that can contaminate surface and drinking water supplies.

The fact that many gas companies—citing confidential business practices—won’t readily disclose their fracking chemicals has also become a public relations issue for the industry. According to an April 2011 report for the U.S. House of Representatives Committee on Energy and Commerce, oil and gas service companies use 750 chemicals during fracking,^4^ some of them—for instance, salt, citric acid, and coffee—fairly innocuous as far as adverse human health effects are concerned, and some not. Naphthalene, xylene, toluene, ethylbenzene, and formaldehyde, for example, each used in a number of proprietary fracking solutions, are known or suspected human carcinogens.^5^ On 17 June 2011 Texas became the first state to require that drillers publicly disclose their fracking chemicals.^6^

## Residential Drilling

The experience of Fort Worth—the epicenter of so-called urban drilling in the United States—offers a glimpse of the emerging issues and public debates around fracking. A fast-growing city of nearly 750,000 people, Fort Worth sits directly atop the Barnett Shale, where nearly 14,000 shale gas wells have been drilled since the late 1990s.^7^ Residents who own mineral rights to their property can sell leases to the gas industry for prices that range from hundreds to tens of thousands of dollars per acre, not to mention 18% or more in royalty payments on production, according to Rolf Hansen, executive director of the Associated Petroleum Industries of Pennsylvania.

But many times mineral rights are owned by people besides the homeowner, which the homeowner may not even have realized at the time of purchase. And property owners who don’t own mineral rights to their land can face a real quandary, says Wilma Subra, president of Subra Company, an environmental group in New Iberia, Louisiana. “You wake up, and a crew is in your backyard drilling a well,” she says—a situation that can pit neighbor against neighbor.

Those crews bring in condensate tanks, which rid the gas of nonmethane hydrocarbons, and they also bring in water and compressor stations that help push the gas through a pipeline. Subra says the condensate tanks emit a number of noxious compounds, including carbon disulfide, which smells like rotten eggs and causes cardiovascular, neurologic, and hepatic effects with chronic high exposure.^8^ What’s more, when compressor stations undergo periodic maintenance, their gas contents are either flared or vented directly into the air, increasing the risk of exposure for local residents, pets, and livestock, according to Subra. Meanwhile, she says, the drilling goes on night and day: “You’re dealing with lights and noise and trucks coming in and out, and you have virtually no authority over the situation whatsoever.”^9^^,^^10^^,^^11^

Shale gas drilling is often accompanied by anecdotal reports of health problems. Some residents living near these facilities complain of headaches, diarrhea, nosebleeds, dizziness, blackouts, muscle spasms, and other problems, as portrayed vividly in the documentary film “Gasland,”^12^ which was nominated for an Academy Award in 2010. But detailed studies into these adverse health effects are lacking, and research conducted to date has yielded conflicting results.

Consider the experience of residents from DISH, Texas,^13^ who live amid a gas production site on the Barnett Shale. After they complained of noise, odors, and vibrations from the drilling sites,^14^ as well as illnesses among community members,^15^ two organizations came to sample the local air. One of them, a Washington, DC–based environmental group called Earthworks, detected carbon disulfide along with dimethyl disulfide and methyl ethyl disulfide—which are both skin, eye, and respiratory irritants—at levels above air quality standards set by the Texas Commission on Environmental Quality (TCEQ).^16^ The other consulting group, Wolf Eagle Environmental of Flower Mound, Texas, also detected elevated levels of benzene (a known human carcinogen), xylenes, and naphthalene.^14^^,^^17^^,^^18^^,^^19^

Those findings drew the attention of the Texas Department of State Health Services, which conducted its own analysis of blood samples from DISH residents. But the state’s investigation showed that blood levels of numerous chemicals in DISH residents weren’t any higher than those predicted for 95% of the U.S. population. Benzene, in particular, was elevated only in the blood of smokers from DISH, and cigarette smoke is a known source of this chemical. The state investigators suggested the chemicals in the blood of the DISH residents likely came from exposure to consumer products or to disinfection by-products in drinking water. But they also pointed out a key limitation to the study: as a one-time sampling event, it wouldn’t have detected chemical spikes that might result from changes in temperature, wind-speed, or variations in nearby natural gas operations.^20^

## The Need for Health Effects Studies

Bernard Goldstein, a professor in the Graduate School of Public Health at the University of Pittsburgh, says published epidemiologic studies relating shale gas production to health are virtually nonexistent. And that makes it challenging to scientifically validate anecdotal reports of health outcomes, he says. “We get lots of complaints from individuals about air quality near these fracking operations,” Goldstein says. “They smell things that don’t make them feel well, but we know nothing about cause-and-effect relationships in these cases.”

In Goldstein’s view, efforts to expand shale gas production are sure to be accompanied by the appearance of disease clusters that community activists will blame on the industry. “You’ll get clusters of cancer or autism—you name it—and then people will say, ‘This never happened before they started drilling here,’” he says. “And then we’ll have to investigate these clusters using retrospective methods that almost always generate unsatisfactory results.”

To avoid that problem, Goldstein recommends that shale gas expansion be accompanied by prospective health research, including baseline disease surveillance and environmental monitoring. That way, if clusters do appear, he says, pre- and postdrilling data can be compared.

**Figure:**
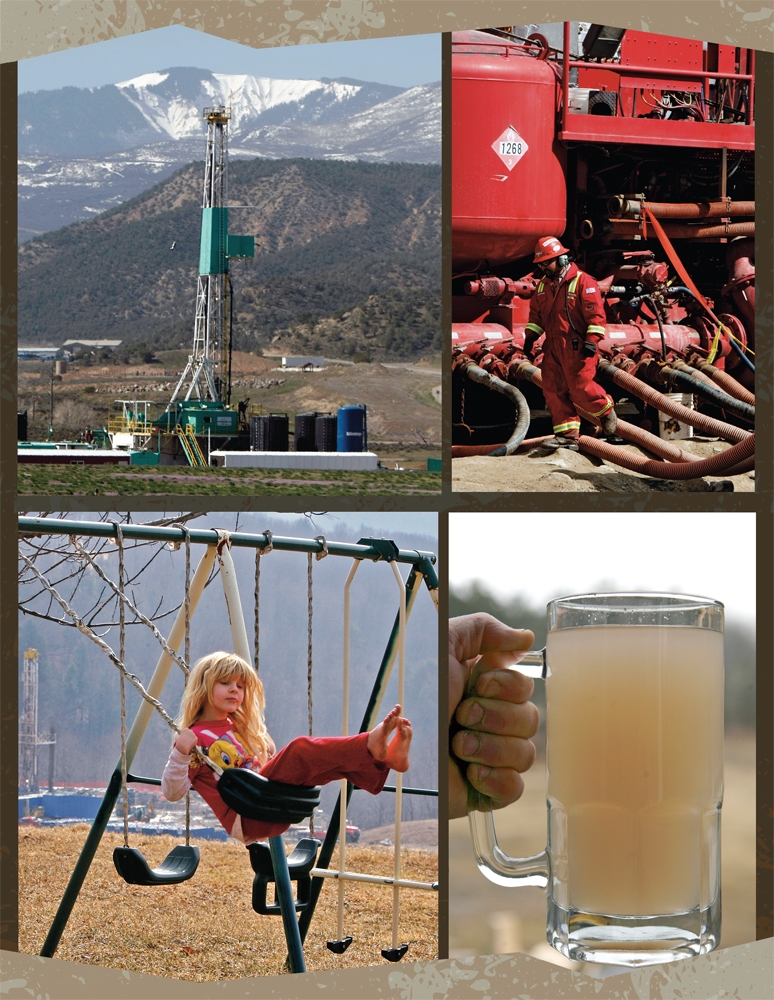
Although shale gas offers many benefits as a fuel and a source of jobs and revenue, the process of extracting it from the ground has drawbacks for surrounding communities. (Clockwise from top left) A natural gas well pad sited near Rifle, Colorado; a worker at a fracking site in Rulison, Colorado; a glass of water taken from a residential drinking water well in Dimock, Pennsylvania, after fracking began nearby; a Dimock backyard overlooking a natural gas well. © AP Photo/David Zalubowski, © Tim Shaffer/Reuters.

That research should also engage the community, Goldstein says, because statements attesting to the safety of shale gas development made by industry and regulatory agencies lack credibility in the face of a growing litany of accidents and contamination problems. For instance, on 20 April 2011 (ironically, one year to the day since the BP *Deepwater Horizon* oil well blowout in the Gulf of Mexico), a natural gas well operated by Chesapeake Energy blew out in Bradford County, Pennsylvania—about 260 miles from Pittsburgh—and spewed 35,000 gallons of wastewater and natural gas into the air for 16 hours, leading to more than a dozen residential evacuations.^21^ Many other incidents cited by state regulators against the industry, including explosions, wastewater spills, and illegal discharges, are described in *Fractured Communities*, a report published by the environmental group Riverkeeper.^22^

Only one site-specific study of potential health problems from natural gas development has been attempted so far. Commissioned by the Garfield County [Colorado] Board of County Commissioners, the study investigated potential adverse health effects of a proposed 200-well natural gas operation in Battlement Mesa, home to roughly 5,000 people. Investigators from the Colorado School of Public Health, who performed the study, concluded that community residents—many of them living within 600 feet of the proposed well pads—might experience chemical exposures, accidents resulting from industry operations, and psychologic impacts such as depression, anxiety, and stress.^23^

The investigators offered more than 70 recommendations for minimizing those impacts. But although the study produced a draft document^24^ and two rounds of public comment, the report was never finalized. That’s primarily because community members and Antero Resources—the company behind the proposed development—disagreed over its conclusions.

“In large part, the key differences of opinion revolved around just one part of the study, which was the human health risk assessment,” says Jim Rada, public information officer for Garfield County Public Health. “The Colorado School of Public Health used screening-level risk assessment methods developed by the Environmental Protection Agency [EPA] that the industry felt were too conservative and that some community members felt weren’t conservative enough.”

## Regulatory Uncertainty and Air Pollution

The EPA has limited regulatory authority over shale gas development. When drafting the Energy Policy Act of 2005,^25^ Congress exempted hydraulic fracturing from the Safe Drinking Water Act,^26^ which regulates chemical “injections” into the ground. Mike Nickolaus, special projects director for the Ground Water Protection Council, an Oklahoma City–based association of state regulators, says the Safe Drinking Water Act was originally intended to cover long-term underground injections like those used for chemical disposal or for enhanced recovery in the oil and gas industry. However, fracturing injections typically occur over the span of a day, during multiple daily injections months, or even years apart.

The EPA’s authority over air emissions from shale gas development also is limited, says Joe Osborne, legal director of the Pittsburgh advocacy organization Group Against Smog Pollution. According to Osborne, EPA regulations dictated by the Clean Air Act address major sources of air pollution (typically 10–250 tons per year, depending on the pollutant) or in some cases, aggregations of smaller sources with similar characteristics.^27^ Individual “emission units” within a shale gas production field—including drill rigs, condensate tanks, compressors, and other equipment—rarely generate enough pollution on their own to be considered major sources, Osborne says. It’s possible the entire operation is a major source, but confirming that requires complex, site-specific investigations that states tend to avoid, he says.

What this often means is that companies running these emission units don’t have to report their emissions or even tell state regulators the units exist. But these units can emit substantial amounts of air pollution. In its permit review of a compressor operating in German Township, dated 12 November 2010, the Pennsylvania Department of Environmental Protection lists potential emissions amounting to 73.5 tons per year of nitrogen oxides (NO_X_) and 19.36 tons per year of volatile organic compounds (VOCs), both well above the EPA’s 10-ton threshold for a major source under the Clean Air Act.^28^ Both NO_X_ and VOCs contribute to ground-level ozone, an air pollutant with significant respiratory and cardiac health effects.

**Figure:**
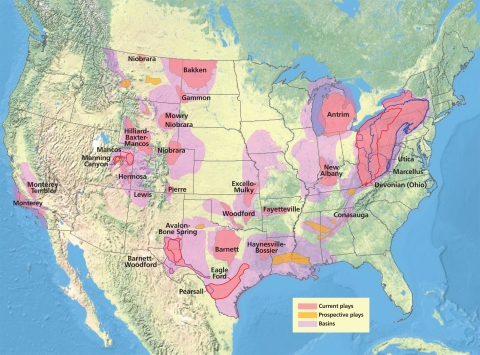
Natural gas exploration, lower 48 states, as of 9 May 2011. “Basins” are sedimentary rock formations known to contain shale gas. “Plays” are sites where developers are actively looking for natural gas in shale rock. The Energy Information Administration estimates that technically recoverable shale gas reserves have the potential to satisfy domestic consumption in the United States for more than 30 years. Source: Energy Information Administration, http://tinyurl.com/3uhqnh5.

Yet the evidence linking shale gas development to high ozone levels is sparse. According to the TCEQ, extensive investigations around the Barnett Shale show “no immediate health concerns from air quality in the area,” despite extensive production.^29^ TCEQ spokesman Terry Clawson says the commission has analyzed nearly 700 field samples collected during eight “mobile monitoring trips” since 2009, using continuous sampling performed by gas chromatography. Those investigations and others didn’t reveal short- or long-term exceedances for any of the 46 measured compounds, including benzene, he says.

Moreover, ozone levels have been trending downward in the Dallas–Fort Worth area despite a dramatic increase in shale gas operations on the Barnett. David Brymer, director of the TCEQ Air Quality Division, says oil and gas development in the Barnett Shale area has resulted in an increase of ozone precursor emissions although ozone levels themselves have generally improved in the area. He attributes this phenomenon to the state’s NO_X_ control strategies and also to prevailing winds that carry shale gas emissions away from the Dallas–Fort Worth area.

Many environmentalists who link shale gas to high ozone levels cite data from Sublette County, Wyoming, a rural area with extensive natural gas development. Winter ozone levels in Sublette County routinely spike over the EPA’s 8-hour ozone standard of 75 ppb—for instance, exceeding the EPA standard 13 times over the period 14 February–15 March 2011.^30^ At times, air quality in Sublette County is even worse than it is in Los Angeles, an anomaly Keith Guille, a spokesman for the Wyoming Department of Environmental Quality, attributes to the area’s oil and natural gas development. “[These industries are] certainly our biggest sources of VOCs and NO_X_,” he says.

However, Guille cautions that other factors also contribute to the region’s ozone problem. Ozone is created when VOCs and NO_X_ interact with sunlight. Uniform snow cover reflects and concentrates sunlight, so it might help boost ozone levels, as might temperature inversions that trap the pollutant in mountain basins, Guille says. Moreover, he says, VOC and NO_X_ emissions in the area have declined steadily since 2007, which suggests snow cover and other variables play key roles in the region’s more recent air quality decline. Unfortunately, the Wyoming Department of Environmental Quality has no pre-2005 baseline ozone monitoring data, so it is difficult to assess the long-term influence of gas development on the region’s air quality, Guille says.

## Questions about Water Quality

Environmental threats from shale gas development aren’t limited to air quality—water pollution is also a serious concern. Each fracking event requires 2–4 million gallons of water. The EPA estimates 35,000 wells undergo fracking annually in the United States, requiring the amount of water consumed in a year by some 5 million people.^31^ Most of that water arrives and leaves by truck, which strains road systems, unnerves local residents, and boosts the risk of automobile accidents, according to Subra. What’s more, chemicals make up 0.5–2.0% of what’s found in fracking fluids—a small percentage of the total that can nevertheless add up to hundreds of thousands of gallons injected directly into the ground.

The natural gas industry insists fracking has never contaminated groundwater. Groundwater resources, officials purport, are protected by thousands of feet of intervening rock between aquifers and shale gas deposits located deeper underground. Could the fracking process connect these two geological layers, allowing chemicals and drinking water to mix? Nickolaus says probably not. “The process is engineered to avoid that,” he says.

Robert Jackson, a professor of environmental sciences at Duke University, also says fracking fluids likely won’t penetrate upward from shale into groundwater. But he adds that natural gas components—particularly methane—can leak through poorly constructed wells into an aquifer.

In a recent study, Jackson found what he describes as “systematic evidence for methane contamination of drinking water associated with shale gas extraction” in aquifers overlying the Marcellus and Utica shale formations in northeastern Pennsylvania and upstate New York.^32^ Jackson sampled 60 residential drinking water wells for dissolved methane levels and found that, on average, wells near active drilling sites were contaminated with methane at levels 17 times higher than those found in wells in areas without drilling. What’s more, the average methane level from residential wells near drilling sites—19.2 mg/L—was within the defined action level of > 10 mg/L but < 28 mg/L recommended for hazard mitigation by the U.S. Department of the Interior.^33^ The maximum value of 64 mg/L constituted a potential explosion hazard.^32^

**Figure:**
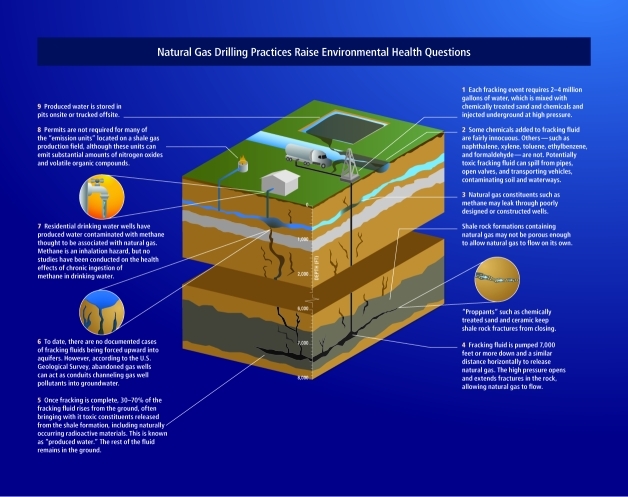
Natural Gas Drilling Practices Raise Environmental Health Questions Adapted from original artwork courtesy of the Checks and Balances Project.

More recently, as reported by the Associated Press on 17 June 2011,^34^ officials from the Pennsylvania Department of Environmental Protection began investigating reports of methane contamination in seven wells located in Lycoming County, near a drilling operation run by XTO Energy Inc., a subsidiary of ExxonMobil Corporation. XTO has since halted its drilling operation and provided residents with bottled water.

Methane may be explosive at high levels, but are low levels in drinking water toxic? That’s not entirely clear, according to Wendy Heiger-Bernays, an associate professor at the Boston University School of Public Health. “We normally think of methane toxicity in terms of inhalation,” she says. “And by that route, we know it can displace oxygen, which creates an asphyxiation hazard, resulting in headaches and nausea and death at much higher concentrations. But we know virtually nothing about how methane might affect people who ingest it.”

Heiger-Bernays says methane is likely to be less toxic in isolation than as part of a chemical mixture, such as might be found in contaminated drinking water. For instance, by interacting with chlorine, methane might produce chlorinated hydrocarbons that are known to be toxic by ingestion, she says. Given that fracking has become more common near populated areas, toxicologic studies of low-level methane exposure in drinking water should be seen as a priority, Jackson adds.

Scott Anderson, who spent many years working for the oil and gas industry before becoming a senior policy advisor in the Environmental Defense Fund’s energy program, says the emphasis on fracking’s groundwater safety record ignores a much larger environmental problem: surface spills involving “produced water” that rises from the shale once fracking is completed. The amount of produced water generally ranges from 30% to 70% of what was injected into the ground. This material contains not just fracking chemicals but also enormous amounts of salt, some radionuclides, heavy metals, and other contaminants drawn to the surface from the shale formation below. “It’s bad, bad, stuff,” says Anderson. “So when industry argues that fracking hasn’t caused any groundwater problems, what’s overlooked are the hundreds of instances in which spills related to surface operations have contaminated other water supplies.” Riverkeeper lists many such instances in its *Fractured Communities* report.^22^

In September 2010 the EPA—charged by Congress to study the potential impacts of fracking on drinking water resources—asked nine leading fracking service providers to disclose the composition of their fracking fluids as well as any health effects data and information about standard operating procedures.^35^ All but one of the companies complied fully; when subpoenaed, the ninth, Halliburton, also agreed to provide the information.^36^ In February 2011, the EPA released a draft plan for its study.^37^ The plan includes proposed investigations into how produced water can leak from onsite storage pits as a result of improper construction, maintenance, or closure. Two prospective case studies will be conducted in Louisiana and Pennsylvania, while five retrospective studies will be conducted in North Dakota, Texas, Pennsylvania, and Colorado.^38^

## Moving Ahead

Is the country ready for full-tilt fracking? That’s debatable. Shale gas clearly has its benefits: it’s domestically produced, it generates jobs and billions of dollars in revenue, and it could arguably lower the country’s greenhouse gas emissions. Natural gas emits less carbon dioxide per unit burned than coal and gasoline, but in its unburned state, it is itself a more potent greenhouse gas than carbon dioxide. Because of that, experts debate its climate benefits.

Some states, among them Texas and Pennsylvania, have embraced shale gas as a revenue source that might boost sagging economies. Other states are taking a more cautious approach. For instance, Maryland tried to impose a two-year moratorium on fracking that failed in the state’s Senate. Instead, Maryland governor Martin O’Malley issued an executive order requiring additional studies of the fracking process, specifically on the Marcellus Shale in western Maryland.^39^ No drilling permits have been authorized in Maryland so far. Meanwhile, the New York State Assembly passed a bill to extend a moratorium on fracking in the Marcellus through June 2012. At press time, regulators were awaiting results of the state’s revised Supplemental Generic Environmental Impact Statement on fracking,^40^ and it was uncertain whether the bill would be passed by the state Senate. New York governor Mario Cuomo was expected to seek to lift the moratorium.^41^

“We need to appreciate what we’re getting ourselves into,” says Robert K. Sweeney, chairman of the New York State Assembly Standing Committee on Environmental Conservation. “It’s not just the pumping of chemicals into the ground or the air pollution, it’s also the effect on quality of life—something as simple as truck traffic, which other states didn’t consider when they issued permits. I’d like to see a cost–benefit analysis that considers the upside of fracking—the jobs, the revenues—but also the downside in terms of loss of property values and health impacts. There’s a lot to this issue that argues for taking our time. The gas isn’t going anywhere, so what’s the rush? If we do it, we should do it right.”
